# Characterizing Managerial Decision Making in Public Hospitals: A Case Study from Romania

**DOI:** 10.3390/healthcare12232395

**Published:** 2024-11-29

**Authors:** Carmen Marinela Cumpăt, Daniela Huțu, Bogdan Rusu, Muthana Zouri, Nicoleta Zouri

**Affiliations:** 1 Department of Medical Specialties III, “Grigore T. Popa” University of Medicine and Pharmacy, 700115 Iasi, Romania; marinela.cumpat@umfiasi.ro; 2 Faculty of Economics and Business Administration, Alexandru Ioan Cuza University, Carol Boulevard, nr. 11, 700506 Iasi, Romania; 3 Faculty of Industrial Design and Business Management, “Gheorghe Asachi” Technical University of Iași, 700050 Iasi, Romania; bogdan.rusu@academic.tuiasi.ro; 4 Pilon School of Business, Sheridan College, 4180 Duke of York Boulevard, Mississauga, ON L5B 0G5, Canada; muthana.zouri@sheridancollege.ca; 5 School of Engineering Technology and Applied Science, Centennial College, 941 Progress Ave., Scarborough, ON M1G 3T8, Canada; nzouri@centennialcollege.ca

**Keywords:** managerial decision making, decision-making triangulation, autoethnographic methodology, managerial ethics in healthcare, resource allocation in hospitals, strategic healthcare reforms

## Abstract

Background/Objectives: Our study investigates the primary characteristics of managerial decision-making processes in the public hospital units in Romania, particularly in the Northeast region. This research aims to delineate the decision-making model applied by managers in these units, considering the multitude of legislative, economic, technical, ethical, and organizational changes prompted by the pandemic. Methods: A mixed-method research approach was utilized, combining semi-structured interviews and autoethnography, to capture experiences, attitudes, perceptions, motivations, and ethical considerations of decision-makers within the healthcare system. Results: The findings revealed that managerial decisions in public hospitals were influenced by unique elements such as the vulnerability and support needs of patients, the absence of a clear hierarchy, the personalized nature of healthcare services, the complexity of care processes, and the use of advanced technology. External factors, notably political and economic influences, alongside internal ethical dilemmas, significantly impacted decision making. Conclusions: This study identifies the reliance on evidence-based decision making and a consultative managerial style as key to addressing these challenges. This research contributes theoretically by comparing decision-making models and practically by identifying a decision-making model that includes forms, techniques, and tools that could guide managers in decision making in Romanian public hospitals.

## 1. Introduction

The healthcare system encompasses the provision of all health services, involving medical and auxiliary staff, institutions, and organizations dedicated to ensuring universal access to high-quality medical care. Due to the complexity of the healthcare system, decision making in this field is a challenge for management. On one hand, medical professionals must quickly decide on the appropriate course of action when people’s lives are at risk, and on the other hand, managers of hospital units must provide quality care with limited resources while simultaneously generating significant and sustainable revenues. In this context, decision-makers are compelled to comply with existing legislation and to understand and effectively manage competition [1].

Hospital unit managers operate within an increasingly comprehensive and multilateral system. Taking into account the multitude of legislative, legal, economic, technical, ethical, and organizational changes, the managerial decision-making process has a direct impact on the institution’s performance, its staff, and the community it serves, being influenced by a series of other factors that could result in financial success, patient satisfaction, and the long-term viability of the organization. Although decisions are made at all managerial levels, those made by top-level managers have a wider applicability, affect more people, and have a greater impact [2,3].

After the 1990s, Romania’s healthcare system was involved in a reform process, when it changed its organization and operation mode, with significant efforts being made to make it more efficient and to offer the best possible services to the population. Currently, the performance of Romania’s healthcare system and public hospitals is not a major national objective; it is not evaluated through a comprehensive model, but mainly conditioned by the manager’s expertise. The performance indicators of the Romanian healthcare system, although improving, remain below the average levels of the European Union, reflecting a deficient management unable to meet the increasingly diverse health needs of the population [4].

In this sense, the performance of public hospitals is influenced by elements such as managers’ reluctance to use modern management techniques and methods for evaluating employee performance, medical technology in hospitals, and the quality of services. At the same time, there is a lack of managerial vision to use additional sources of funding, such as sponsorship contracts, hospital space rentals, medical equipment rentals, medical research contracts, etc. Despite these limitations, there is increased pressure on managers of public hospital units to use available resources as efficiently as possible to achieve remarkable results. In addition, they are forced to ensure high-quality care at a lower and more competitive cost and to achieve a performance and offer medical services similar to hospitals abroad, and are constrained by often-insufficient material resources, political involvement, economic uncertainties, as well as an arsenal of norms and regulations that involve lengthy procedures [5].

We consider that the efficiency of hospital activities and the increase in their performance are closely related to the way in which the manager adopts strategic decisions. Such decisions may concern the recruitment of employees, the acquisition of new technological elements, the allocation and use of financial resources, etc. Within public hospital units, decision-makers must decide how to guide and organize the institution and how to control the processes in the system, making decisions frequently based on collected information. The decisions made by managers focus not only on providing the best services to patients but also on meeting the performance objectives set. Ultimately, the performance of the hospital unit is affected by the decisions adopted by its management [6].

Healthcare decision-makers engage in three distinct types of decision making: public policy decisions, which define the range of services to be provided; clinical policy decisions, which determine the recipients of clinical services; and administrative policy decisions, which address the allocation of service locations and the framework for their support and management [7].

Healthcare managers establish the strategic direction of their institutions through critical decisions in key areas, including staff recruitment and training, technology acquisition, and the allocation and management of financial resources. Regularly tasked with decision making based on systematically gathered data, managers must determine how to administer, guide, and organize personnel, manage operational processes, and facilitate decision making within their teams. These managerial decisions extend beyond the immediate objective of delivering high-quality patient care; they are also integral to achieving the institution’s established performance benchmarks.

In this context, the objective of this study is to identify and describe the specific characteristics that define the managerial decision-making model used in public hospital units in Romania, with a particular focus on the Northeast region of the country. To meet this objective, a mix of qualitative research methods (through triangulation) was used, namely the survey method based on interviews and the autoethnography method. In this sense, this paper theoretically contributes to the development of the literature on a little-explored phenomenon regarding how managers of public hospital units in Romania make decisions and the significance they attribute to managerial activity, and also makes a comparison of the decision-making models used in hospital units. Thus, we identified opinions, attitudes, perceptions, and motivations regarding the factors that influence the managerial decision-making process, what are the criteria for adopting certain decisions, what are the sources of evidence, what are the stages gone through for making a decision, how managers of public hospital units in Romania use information flows, who are the people they consult with, if they consult, as well as the impact that ethical aspects have on the managerial decision-making process [8].

This paper is organized to align with the stated research objectives. Section 2 outlines this research’s methodological framework, providing a comprehensive overview of both theoretical and practical aspects that align with the research aim and the targeted data. It details each stage of implementing the qualitative methods employed. Section 3 presents the findings, initially through an analysis of semi-structured interviews, followed by insights gathered from autoethnographic techniques. Section 4 is dedicated to discussing these results, comparing insights gained from both methods, and contextualizing them within relevant scholarly literature. Lastly, Section 5 concludes this study by emphasizing the critical findings, particularly focusing on the managerial decision-making model applied in Romanian hospital units [9].

## 2. Materials and Methods

**Figure 1:**
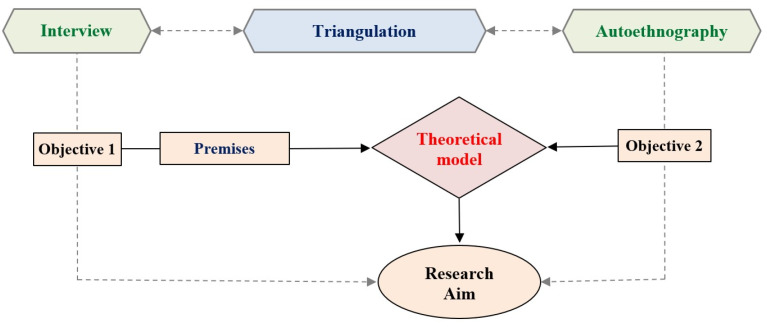
Research methodology scheme.

In this research, the purpose of using interviews was to gain a deep understanding of managers’ experiences regarding how they make decisions and the significance they attribute to managerial activity. The choice of mixed research methodology is justified by the nature of the phenomenon studied, which reflects a complex reality and depends on how managers relate to the challenges this reality implies. Subsequently, to improve the reliability and validity of the information, analyses, interpretations, and conclusions collected through the survey based on interviews, we chose to compare the data obtained through the analysis of interviews with data generated by autoethnography, a comparison that actually involves data triangulation. Thus, with the help of the two qualitative methods, the survey method based on interviews and autoethnography, comparisons could be made between the researcher’s own experiences, in his capacity as a manager, and the analysis carried out on the data collected through interviews, thus allowing through induction the shaping of a theoretical model regarding the decision-making process of managers in Romania. In other words, the premises extracted from the qualitative analysis (based on the interview) form the basis for formulating the theoretical model, which is validated with the help of autoethnography. The methodological scheme of the present research is shown in Figure 1.

Aligned with the fundamental objective, this research’s operational framework is outlined, including the formulation of specific theoretical and methodological objectives. The first specific objective involves generating a theoretical model regarding the characteristics that define the decision-making process at the level of public hospital units in Romania. The second specific objective aims at verifying the validity of the theoretical model defined in the first stage of this study.

Given the opinion that findings resulting from two or more methods increase the confidence that the results are valid and do not represent a methodological artifact [10], the two qualitative methods were used, whose mixing was made possible through triangulation. Thus, deducing premises from the interview analysis forms the basis for formulating the theoretical model of this research, which is validated through autoethnography.

### 2.1. Study Design and Population

**Figure 2:**
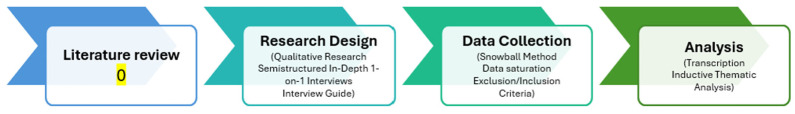
Study design.

We conducted qualitative research using semi-structured, one-on-one interviews with 10 managers of public hospitals in Romania to gain in-depth insights into their decision-making processes. This design enabled a comprehensive exploration of the participants’ perspectives and experiences. Sampling was carried out through a combination of purposive sampling, to ensure participants met the criteria of being public hospital managers, and the snowball method, which facilitated the identification of additional qualified participants through referrals. The process continued until data saturation was achieved, meaning no new or relevant information emerged from additional interviews, thus signaling a thorough understanding of the subject. This approach aligns with qualitative research standards, emphasizing the depth and richness of the collected data over sample size. The interview guide was developed to cover key topics relevant to managerial decision making and was validated by two experts from the Alexandru Ioan Cuza University of Iasi. This study was conducted between 2018 and 2022. The study design is illustrated in Figure 2, which outlines the methodological framework and key steps followed in this research.

For the construction of the interview guide, all changes deduced from the pretesting stage were taken into account. In this sense, the interview guide was structured in two parts, the first part including questions related to the personal characteristics of the interviewed managers, and the second part consisting of 15 open-ended questions regarding the issues of the current research (see Supplementary Materials S1).

### 2.2. Interview Procedures and Data Collection and Analysis

In this stage of the research, data on the managerial decision-making process at the level of hospital units in Romania were collected through the use of the interview-based survey method. Among the types of interviews existing in the literature, we chose the semi-structured in-depth interview. The choice of this qualitative research method is justified by its use for deeply probing opinions, attitudes, and motivations of managers to identify possible unconscious information [11]. In other words, the aim was to identify opinions, attitudes, perceptions, and motivations of the participants invited to discuss factors that influence the decision-making process, what are the criteria for adopting certain decisions, what are the sources of evidence, what are the stages gone through for making a decision, how information flows are used, who are the people they consult with, if they consult, as well as the impact that ethical aspects have on the managerial decision-making process.

A first step in the analysis of the interviews was their transcription, resulting in approximately 162 pages of transcription. Thus, each interview was transcribed separately, and each line was numbered. Then, the analysis of the information was started, which, according to [12], is the directly acquired information, in fact, the information obtained from the interviewees. Based on this information, the first stage of data analysis is approached, namely the stage of open or initial coding [13], which involves the systematic reading of the data and making memo-type notes. In other words, each interview was read line by line, on the margins of which the researcher made annotations and reflected on the meanings of different discursive elements of the participants [14]. Ref. [12] considers this step the beginning of the interpretative stage.

Subsequently, the data thus obtained were synthesized and processed using thematic–categorical content analysis, which presupposes a text analysis technique. In this context, content means words, images, meanings, symbols, ideas, etc. [15]. This method involves a series of steps: coding each statement taken from each interview; classifying the data based on the themes and/or concepts prevalent in the discourse of the interviewed persons; axial coding, which involves classifying themes and/or concepts into semantic categories; and selective coding, which aims to unite all themes and semantic categories into a single fundamental category or domain, which will underlie the definition of the theoretical model.

### 2.3. Ethical Considerations

The interview guide was accompanied by an informed consent form in which relevant information about this study’s proceedings was presented: the research theme, requested information, time to be allocated to the interview, risks, benefits of participating in this study, the possibility of withdrawing at any moment from this study, as well as maintaining anonymity and respecting personal data in accordance with European legislation in the field.

All data were treated confidentially and pseudonymized with due care throughout the entire research. Depending on our research questions, the recorded interviews were only listened to and typed by the researcher. The interviews were transcribed anonymously. The audio recordings and transcriptions were recorded in a secure manner, and these files were saved on a password-protected personal computer. This study was conducted on a voluntary basis, and no compensation was offered. This research was approved by the Ethics Committee from the Clinical Rehabilitation Hospital Iaşi on 13 December 2018. The approval does not have an approval code as the process in Romania does not always provide one; instead, it has an approval date.

To minimize potential researcher bias, the interview guide was subjected to expert validation. During the interviews, the researcher refrained from intervening in participants’ responses, actively listened, and maintained an impartial stance, avoiding any validation of interviewees’ statements. This approach helped ensure objectivity in data collection and analysis.

### 2.4. Autoethnography

In the last decade, there have been many publications on the topic of autoethnography, covering various disciplines, including those in the health field [16]. However, this methodology is still very new in the health sector in general, and in hospital management in particular.

Autoethnography is a qualitative research method defined by [17] as the method “that uses the researcher’s personal experience to describe and critique cultural beliefs, practices, and experiences”. Autoethnography is a form of ethnography in which the researcher becomes the subject of study to understand a cultural experience [18]. Ref. [19] considers that one’s own experiences are spaces for exploration, examination, and representation in qualitative research. In addition to highlighting personal experience, the researcher analyzes these experiences using a set of theoretical and methodological tools, as well as specialized literature in the field. Therefore, the researcher contextualizes their own personal experiences and those of others within the socio-cultural context to reveal the meanings of the theme analyzed. Thus, the social and cultural context can be understood from the perspective of a group of individuals with similar experiences [20].

As a qualitative methodology, autoethnography offers several advantages. One primary benefit is that it provides access to the researcher’s inner world, thereby generating rich, in-depth data [21,22]. Another advantage is the accessibility of data, as the researcher uses their own experiences as a source for analyzing a phenomenon. However, this reliance on personal narrative also presents a limitation, as it can constrain the scope of the research findings. Additionally, autoethnography holds the unique potential to engage and resonate with external stakeholders, encouraging them to reflect on and empathize with the presented narratives [23]. By examining cultural or social accounts of experience, autoethnography serves as a valuable research approach, uncovering previously unrecognized realities.

In the present research, the justification for choosing this method stems from the professional position of the researcher, who has held the position of manager of a public hospital for over 14 years and leadership positions in public hospital units in Iași County, Romania, for over 20 years. Consequently, the phenomenon studied, namely the specifics of the managerial decision-making process in the sphere of public hospital units in Romania, is very familiar to the researcher.

The researcher’s personal memory was the most important source of data, creating an autobiographical chronology of events, lived experiences, and decisions made as a manager. Systematically, self-observation, introspection, and self-analysis were conducted through recording the behaviors, thoughts, and emotions of the researcher. In this context, the researcher was interested in presenting his reality alongside that of the interviewees and to see what version of reality would emerge from the analysis. The researcher thus brought into the analysis interpretive frameworks, concepts, and knowledge regarding the managerial decision-making process that are part of his experience and professional positioning.

## 3. Results

### 3.1. Characteristics of Study Participants

The 10 interviews were conducted in the Northeast region of Romania with managers of public hospital units in the Counties of Iași, Suceava, Neamț, Focșani, and Vaslui. The interviewed managers were aged between 35 and 63 years, of which six were male, and four were female, with professional training in the medical (five respondents), economic (three respondents), or legal (two respondents) field. Out of all respondents, seven had higher education, and the other three had postgraduate studies. Also, all 10 interviewed individuals had undergone management training. Regarding the number of years of work in a healthcare unit, six of the respondents reported seniority between 5 and 15 years, and the remaining four had between 20 and 25 years of work. According to the number of years they had held a leadership position in a hospital unit, the 10 interviewed individuals were distributed as follows: one person with 1 year as manager; three respondents with 2 years in the manager position; one person with a seniority of 5 years; three of the interviewees had been managers for 8 years; and only two of the respondents had a seniority of 9 years as managers in a hospital unit.

### 3.2. Results of the Thematic Analysis of the Interviews

The results obtained from the thematic analysis of the data collected through the interview survey highlighted five essential dimensions, as well as the corresponding subdomains, of the decision-making process of the interviewed managers (see Supplementary Materials S2). The five categories refer to: (1) areas of managerial decision; (2) unique elements of hospital units; (3) factors influencing managerial decision; (4) sources of evidence in the managerial decision-making process; and (5) models of managerial decision making. Additionally, each domain and subdomain are elaborated upon in the subsequent sections and synthesized with the most pertinent quotes from the interviews, which are presented in Supplementary Materials S3–S6.

#### 3.2.1. The Domain of Managerial Decision

Regarding the first identified category, namely the domain of managerial decision, the findings from the content analysis of the interviews showed that the decision-making process is a matter of great responsibility for the managers of public hospital units in Romania. The interviewed managers stated that they usually make decisions based on collected information, decide how to administer, guide, and organize others, how to manage processes in the system, and how to help others make their own decisions. At the same time, they highlighted that they are constantly under pressure to make the best use of available resources to achieve the best results for both the unit itself and other stakeholders. In summary, the managers’ responses indicate that the most frequent decisions taken are related to (a) adopting the budget and allocating resources, (b) recruiting, training, and developing staff, (c) acquiring technology, and (d) strategic planning.

#### 3.2.2. Unique Elements of Hospital Units

The analysis of the collected data allowed for the formulation of conclusions related to the unique elements of hospital units and the outline of several related subdimensions: (a) the state of vulnerability and the need for support of patients; (b) the personal and personalized nature of healthcare services; (c) the absence of a clear hierarchy; (d) the complexity of the care process; and (e) the highly advanced technology.

Firstly, the responses of managers during interviews showed that patients are often the most vulnerable, due to the asymmetry of power and information in the relationship between them and the medical staff, being much less capable of acting independently. Moreover, patients are emotionally fragile in the face of detection and communication by the doctor of a medical diagnosis, but also physically weakened by the experience of illness or the effects of treatment. The need for support, the lack of information, and the impact of the diagnosis most often cause them fear, dissatisfaction, and anxiety. At the same time, patients are not content to be passive recipients of healthcare services; they expect to be consulted, informed, and involved in any decisions that have to do with their own health.

Secondly, it emerged that the care process is a complex one, with the interviewed managers emphasizing the special responsibility to compensate for the inevitable asymmetry of power and information in doctor–patient relationships by providing services that are patient-centered, ensuring good communication, understanding, and involvement of the staff.

Thirdly, the responses highlighted deficiencies regarding the existence of a clear hierarchy and the assumption of responsibility by higher hierarchical bodies, in a context where hospital units are facing increasingly significant challenges generated by the lack of staff, the pace of technological innovation, changing patient expectations, and rising costs.

#### 3.2.3. Influencing Factors of Managerial Decision Making

In the interviews, the influence of both external and internal factors in managerial decision making is highlighted. Among the external environmental factors, the political factor was the most frequently mentioned, followed by the economic factor, and to a much lesser extent, the social and geographical factors (which is why they were excluded from the content analysis). The interviewed managers mentioned that the political factor plays an essential role in the hospital units they lead. Some decisions, which involve the presence of multiple actors in their adoption and entail significant changes, are likely to provoke public disputes. In this context, the challenge for managers is to succeed in obtaining political support without losing the support of important groups within the unit and the community, while also being efficient in the decision-making process. Additionally, the economic factor is considered determinant in managerial decision making, as all interviewed managers mentioned this aspect at least once during the interview. They highlighted the restrictive nature of economic and financial resources on the adoption of decisions, which direct, or rather limit, the actions and measures that will be taken, to the detriment of a compromise that, ultimately, has an impact on the population’s health.

Regarding the influence of internal environmental factors, the study results highlighted the presence of aspects related to the moral and ethical side of the decision-making process. The interviewed managers touched on a series of sensitive topics, generated by value conflicts between different stakeholders with different needs, interests, expectations, backgrounds, and personalities, different cultural environments, strategic contexts and/or situational circumstances of uncertainty, pressures from the internal and external environment, insufficient resources, lack of competence and/or experience, and legislative ambiguities. Moreover, the situation generated by the COVID-19 pandemic has revealed the sensitivity and fragility of hospital units that were faced with unknown, unpredictable situations that often involved ethical aspects. For example, medical staff had to choose between adhering to the visiting regulations in therapy and the insistence of relatives who wanted to be given the opportunity to see the patient in a serious condition. Another ethical dilemma presented was choosing between adhering to current protocols that stipulated a certain period of hospitalization and certain criteria that had to be met for discharge and the insistence of the patient who did not want to be discharged because they did not yet have wood at home for heating.

#### 3.2.4. Sources of Evidence in the Managerial Decision-Making Process

The most important sources of evidence in the decision-making process by the interviewed managers include (a) professional experience and (b) the judgment of the members of the executive committee and the members of commissions and committees. Another part mentioned (c) the hospital information system, (d) the management dashboard, and (e) internal evidence and stakeholder expectations observed through participation in on-call reports or meetings in the administrative area. Equally, the interviewed managers considered (f) legislation, reports, and national, regional, or local plans as important sources of evidence. Very few managers mentioned (g) ethics and (h) religious beliefs as sources that guide them in making decisions.

#### 3.2.5. Managerial Decision-Making Models

From the analysis of the data regarding the last category identified in interviews, namely the models of managerial decision making, it emerged that the majority of the interviewed managers use (a) the administrative model, followed by (b) the evidence-based model, (c) the adaptive model, (d) the DECIDE model, and (e) the incremental decision-making model.

Regarding the use of the administrative model, managers emphasized that to ensure that problems are clearly defined and the evaluation of potential alternatives is easier, they opted for choosing the best solution together with their subordinates. Thus, to install a second generator in the hospital, which required interrupting the hospital’s electricity supply with all associated risks, the interviewed manager relied on his subordinates in making the decision: the medical staff, who had to monitor and ensure there was no risk to patients or that the patients were stable at that time, and the administrative staff, who ensured that backup aspects were functional and could intervene at any moment.

One of the examples given that supports the use of the evidence-based model was the acquisition of equipment. All managers who touched on this topic mentioned that in order to make the decision to buy new equipment or replace one that was no longer functional, they first had to have a report from a service company and a memorandum justifying the need for the purchase. The request was discussed in the executive committee and the medical council where the history was evaluated, and it was analyzed to what extent the purchase is implementable and what benefits it could bring to the hospital, both in terms of medical academic reputation and material benefits, such as revenue at the hospital level.

From the responses of the interviewed managers, it was deduced that they make a series of decisions in a rational, thoughtful, calculated, and measured manner. They also employ the adaptive model, which is characterized by flexibility and intuition in making decisions that involve the adoption of urgent measures, such as procuring blood for a patient scheduled for surgery and for whom the blood transfusion center has not supplied the requested blood products.

Another model used that emerged from the analysis of the interviews is the DECIDE model. In making their decision, the managers adhered to the six steps of the decision-making model: Defining the problem, Elaborating the criteria, Considering all alternatives, Identifying the best alternative, Developing and implementing an action plan, and Evaluating and monitoring the solution. One of the interviewed managers faced the departure of the only doctor in a particular specialty, which led to the closure of the department. To avoid losing patients to competitors and to continue offering services within the department, the first phase considered the alternative of entering into a collaboration contract with one of the specialty hospitals in Iasi County and transporting patients by minibus for specific treatments. The procedure was quite difficult to implement, given the distance and the health condition of the patients. In the second stage, recruiting doctors from the Republic of Moldova was considered, but this was unsuccessful due to difficulties in equating their qualifications. In the third stage, efforts were made to provide opportunities and facilities for doctors to motivate them to take up employment. Apartments were purchased with the assistance of the County Council and offered as service accommodations. Additionally, for those who did not mind commuting, a coach was purchased to transport them to work every morning and take them home at 3:00 PM. By choosing the last alternative, a considerable number of doctors were attracted to participate in the competition, thus solving the problem and making the best decision, obtaining positive feedback from both doctors and patients.

The interviewed managers also use the incremental model for decision making, characterized by the nature of political negotiations and the condition of compromises existing among the stakeholders involved in the decision-making process. The situation generated by the COVID-19 pandemic has provided examples where hospital managers used the incremental model in making decisions, being forced at certain times to admit oxygen-dependent patients when their number exceeded the oxygen supply capacity of the hospital’s oxygen station, a fact known both at the unit level and at the level of the County Public Health Directorate.

### 3.3. Defining the Premises Underlying the Theoretical Model

Based on the previously discussed five categories/dimensions, and implicitly the themes identified in the responses of the 10 interviewed managers regarding decision making, it is possible to identify the fundamental category of the current research, namely the peculiarities of the managerial decision-making process in public hospital units in Romania. This underpins the theory of this research, which is based on a series of research premises:

Premise 1: The unique elements of hospital units in Romania influence managerial decisions.

Premise 2: Managerial decisions in hospital units in Romania are influenced by political and economic factors, and ethical dilemmas and values.

Premise 3: Managers of hospital units in Romania practice a consultative managerial style.

Premise 4: Managers of hospital units in Romania make evidence-based decisions.

To enhance the credibility of the research premises reached regarding the peculiarities of the managerial decision-making process within public hospital units in Romania, autoethnography has been used.

### 3.4. Results of Autoethnography

Through autoethnography or the method of personal experience, the researcher has outlined the challenges faced in the capacity of a hospital manager, how actions were taken to solve problems, the factors that influenced decision making, the sources used to gather information, or the people consulted. Moreover, the researcher connected the present with the past, recognized the relationships between the self and others, compared personal professional experiences with those of other managers, and matched the findings from this analysis with ideas and constructs from the specialized literature.

The situation generated by the COVID-19 pandemic represented one of the greatest challenges for managers of public hospital units in Romania and implicitly for the researcher in their capacity as a public hospital manager. In this context, the researcher, as a manager, made a variety of decisions. These decisions are detailed in the following sections.

#### 3.4.1. Compliance with Legal Provisions and the Institutional Hierarchy

In this regard, in the action plan approved and updated at the unit level by decision no. 754/18.10.2021, it was stipulated that “admission to the COVID sector on floors 6, 7, 5, 4, section E is done only after obtaining the approval from the Public Health Directorate (DSP)”, the hospital being nominated as a COVID-19 support hospital by Order 1343/29.07.2020.

The decision to take in patients infected with the SARS-CoV-2 virus involved, firstly, organizing the spaces, as outlined in the updated action plan: “the technical service staff will completely reorganize the space in the shortest possible time in accordance with Annexes III.1, III.2, III.3, III.4 [...] the arranged workspace, clean area [...] will be equipped with a desk, chairs, 1 refrigerator, 1 medicine cabinet, 1 protective equipment cabinet, 1 telephone, 1 computer, 1 printer”.

#### 3.4.2. Recruitment, Training, and Development of Staff

The researcher, in the capacity of a manager, made decisions to ensure the availability of medical and auxiliary staff necessary for providing care to both COVID-19 and non-COVID-19 patients by consulting current legislation, the executive committee, the head of human resources, as well as the epidemiologist.

At the same time, the researcher, in their role as manager, mandated that both the medical and auxiliary staff of the unit must participate in training courses. These courses are to be conducted by the department head at the department level, “identifying specific COVID symptoms, donning/doffing PPE, and dissemination of the PS 16 procedure Managing patients in the context of the spread of infections with the SARS-CoV-2 virus”. To increase protection measures for all staff, it was decided by the department head to conduct “staff training based on a schedule of all SOPs developed by the Hospital Infection Control and Antimicrobial Stewardship Program”.

#### 3.4.3. Adoption of the Budget, Allocation of Resources, and Acquisition of Technology

The decisions adopted in the context of COVID-19 also targeted the identification of financial resources necessary for the acquisition of personal protective equipment needed for efficient prevention and the equipping with medical devices for rapid diagnosis, facilitating optimal medical care to all patients of the unit. In this regard, the researcher, in their capacity as a manager, decided to access non-repayable European funds. Thus, the unit they manage succeeded in implementing between 2020 and 2022 a project co-financed by the European Regional Development Fund through the Operational Programme Infrastructure. The project aimed at the acquisition of medical equipment, namely ventilators, medical monitors, syringe pumps, infusion pumps, ICU beds, and protective and disinfection materials such as gowns, masks, shoe covers, gloves, coveralls, disinfectants, antiseptic products, and germicidal lamps.

#### 3.4.4. Use of Evidence Sources

In deciding to purchase the drugs necessary for the treatment of patients with COVID-19, the researcher, in their capacity as a manager, primarily relied on current legislation and international research studies. According to the action plan, “the treatment used in treating patients with COVID-19 admitted to the Iasi Clinical Recovery Hospital will follow the National Treatment Protocol for SARS-CoV-2 infection approved by Order 533/2021”.

For treating patients infected with the SARS-CoV-2 virus, although the hospital did not have in its portfolio the provision of infectious diagnostic treatment services, the researcher, in their capacity as a manager, decided to enter into a service contract with an infectious disease doctor who “will collaborate with the attending/on-call doctors, as well as with the coordinating doctor of the COVID area for the prescription of specific medication”.

Considering the risks involved in using oxygen for most patients infected with the SARS-CoV-2 virus, in circumstances where the hospital’s infrastructure was not designed for this purpose, the researcher made decisions, in their capacity as a manager, to limit these risks. Firstly, additional oxygen concentration sensors were acquired and distributed in each room designated for treating COVID-19 patients, and secondly it was stipulated in the action plan applicable at the unit level that “staff working in areas where oxygen is administered are required to monitor the O2 concentration sensors and to ventilate the rooms frequently, at least once every 3 h”.

Making decisions in areas such as resource allocation, staff training, budget adoption, strategic planning, or technology acquisition was influenced by economic factors in that financial deficiencies played the most significant role in decision making, which was not always optimal, and by political factors that either constrained or provided opportunities for change in the short or long term.

#### 3.4.5. Values and Ethical Dilemmas

Equally, the focus on values and ethical dilemmas was much more pronounced throughout the COVID-19 period, with the researcher, who serves as a manager, often being put in vulnerable positions from this perspective. In the context of the action command Order no. 9490/04.10.2021, they were forced to “suspend admissions that were not emergencies” at a time when the schedule list was quite large, and patient dissatisfaction regarding access to medical services was increasingly manifesting. Patients who seek care at the unit are generally individuals with chronic diseases and numerous comorbidities, for whom any delay in investigation, prescription, and therapy administration leads to a decreased chance of recovery. Therefore, the patients’ grievances were justified given the healthcare unit’s positioning in the Northeast region of Romania, the personalized nature of the services offered by the unit, the complexity of the care process, and the patients’ need for support.

The researcher recalls specific moments when, as a manager, they had to choose between complying with the law and facing pressures from patients or relatives who saw the hospital as their only salvation. These moments were filled with feelings of compassion, responsibility, emotion, and, importantly, civic and social duty.

Throughout the COVID-19 period, the number of beds allocated to patients infected with the SARS-CoV-2 virus in the unit managed by the researcher as a manager fluctuated both among ICU beds and among the beds belonging to medical departments, as also presented in the unit’s action plan.

Every time, the researcher, as the manager of the hospital unit, had to decide on the organization of the unit by taking into account the decisions made at the county or national level, the permits and approvals obtained at a certain moment, as well as the constraints of time and resources.

## 4. Discussion

In the present research, data triangulation involved comparing the data obtained through interview analysis with the data generated through autoethnography. This validation technique was used to gain more insights into the peculiarities of the managerial decision-making process at the level of public units in Romania, to recognize and eliminate inconsistencies, and to minimize inadequacy. In other words, through data triangulation, we managed to test the consistency of the findings obtained through the two research methods, adding mutual value and thus enhancing the quality of qualitative research [24].

Therefore, in what follows the results of the qualitative analysis are interpreted in relation to the themes identified based on the evaluation of the interviews, in relation to the researcher’s experience as a manager of a public hospital institution, but also in relation to the theoretical concepts and results of other researchers found in the specialized literature.

### 4.1. The Field of Managerial Decision Making Resulting from Research

The information obtained from the interviews regarding the field of managerial decision making has confirmed the initial framework, prefigured by the study of specialized literature. Managers play a decisive role in approving budgets and in allocating resources for information, with the aim of improving quality [25]. Embertson’s study [26] shows that managers of hospital units are the deciding factor in the unit’s strategy and are responsible for the strategic and financial management of the unit.

Refs. [27,28] have highlighted the importance of managerial decisions regarding technology acquisitions, which can have significant effects on the hospital unit. Ref. [29] found that top healthcare managers make decisions regarding the recruitment and training of staff, conducting performance evaluations, promotions, and demotions, designing and organizing the workload, setting standards, guidelines, and interacting with external entities.

The researcher’s experience as a manager of a public hospital unit reinforces and supports this study’s findings. With over 14 years of activity as a manager of a public hospital, the researcher was placed in the position to make simple or complex decisions related to planning, organizing, allocation, acquisitions, personnel, leadership, or control, with short or long ranges of action, flexible or inflexible, and even decisions in crisis situations.

The situation generated by the COVID-19 pandemic is the prime example where the researcher, in their capacity as a manager, had to make decisions in a context filled with gaps, uncertainties, and unforeseen circumstances. These decisions primarily focused on identifying financial resources for the acquisition of personal protective equipment (disposable masks, FFP2/FFP3 masks, coveralls, face shields, shoe covers, etc.), disinfectants, cleaning materials, medications, medical supplies, as well as supplementing oxygen concentrators, cylinders, and oxygen detectors. Another challenge was making the best decisions to train, prepare, and ensure the necessary medical and auxiliary staff for providing medical care to both COVID-19 and non-COVID-19 patients, at the hospital level as well as at the hospitals within the county for which the Public Health Directorate Iasi requested support. Additionally, there was a need to plan and organize spaces, reorganize circuits, work flows, and adapt work schedules.

Consequently, the results obtained from the analysis of data collected through interviews, on one hand, and the theoretical and practical studies found in the specialized literature, as well as the experiences of the researcher as a manager of a public hospital unit in Romania recorded through autoethnography, on the other hand, all highlight that the managerial decisions adopted target four major areas: budget adoption and resource allocation, recruitment, training, and development of staff, technology acquisitions, and strategic planning.

### 4.2. Unique Elements of Hospital Units Emanating from Research

The study conducted by Walshe and Smith [30] and the analysis of the results obtained from conducting interviews provide the opportunity to identify the following unique elements characterizing hospital units: (a) the state of vulnerability and the need for support of patients; (b) the absence of a clear institutional hierarchy; (c) the personal and personalized nature of healthcare services; (d) the complexity of the care process; and (e) highly advanced technology.

Throughout their managerial activity, the researcher reflected on the specificity of the hospital unit they manage, which primarily stems from its social mission to offer recovery services adapted to the specific pathologies of the patients. The process of medical care for patients needs to be complex, supporting patients who place their entire trust in the unit they turn to and expect to find a hospital environment free from hostility, an environment that provides them with physical and psychological comfort and diminishes their state of vulnerability.

People accessing medical services at the unit managed by the researcher are generally older individuals with chronic diseases and multiple comorbidities for whom complex treatments are necessary, along with investigations using the most advanced technological equipment. All these factors imply increasing costs and contribute to the constant pressure for greater funding of the hospital unit, which is often difficult to achieve due to the institutional hierarchy.

The conclusion drawn from corroborating theoretical studies in the specialized literature and the experience gained from the researcher’s activity as a manager is that the personalized nature of healthcare services, the complexity of the care process, the vulnerabilities and need for support and sustenance of patients, the absence of a clear hierarchy, as well as the advanced nature of the technologies used contribute to outlining particularities regarding the decision-making process in public hospital units in Romania.

### 4.3. Influencing Factors of Managerial Decision Making Identified Through Research

The results of the analysis of responses from interviewed managers regarding the implications of politics and economics in the decision-making process at the level of hospital units, as well as the presence of ethical aspects (see Section 4.3), are supported by the specialized literature. In this context, ref. [31] showed that the decisions of hospital managers in European countries like Poland are dependent on the political environment. Similarly, ref. [32] demonstrated that politics, along with national directives, ethics, common knowledge, the economy, and organizational and institutional complexity, are the main contextual factors influencing the decision-making process of resource allocation in the health system of Australia.

The study results from [33] indicated that information on clinical efficacy and economic aspects were considered the most relevant when deciding on investments in new health technologies in European hospitals.

Walker’s study [34] highlights the fact that ethical values form the basis of ethical decisions. These fundamental values include trust (including honesty), respect, care, responsibility, justice and fairness, civic virtue, and citizenship. Similarly, ref. [35] explores managers’ experiences and views on ethical issues in the decision-making process. In this context, he observes that a rigorous qualitative analysis of experiences, strategies, and responses observed to ethical problems led to the classification of competencies associated with judgment, integrity, courage, and humanity.

Over the 14 years of managing a public hospital unit, the researcher was often in situations where they had to understand the political dimensions of problems and proposed solutions, thus managing to better anticipate both short-term constraints and long-term change opportunities. Equally, the financial deficiencies they frequently faced have limited the adoption of certain decisions that could have contributed to improving the quality of medical services offered by the unit they manage.

During the COVID-19 pandemic, the researcher, as the manager of a public hospital unit designated as a COVID-19 support facility, had to manage vulnerable situations that involved ethical dilemmas and values. Thus, they were forced on one hand to face pressures from relatives and the local press accusing the unit of not providing information about hospitalized COVID-19 patients, and on the other hand to comply with current legislation. Moreover, the treatment of patients infected with the SARS-CoV-2 virus by medical staff from specialties other than infectious diseases, the isolation and hospitalization of asymptomatic patients, as well as providing medical care to minors infected with COVID-19 allocated to the hospital managed by the researcher without having a pediatric department represented other ethical dilemmas. Throughout this period, the researcher, in their capacity as a manager, made decisions guided by a set of ethical principles and values. In this sense, to show respect and care for patients, they concluded contracts with a Catholic priest and Jewish Rabbi for patients in critical condition and for whom the family requested religious support.

The conclusion emerging from the previously shared experiences is that the political and economic factors are very important in the managerial decision-making process at the level of public hospital units in Romania, and that managers need to be aware of their influence in order to make the best decisions [36]. Moreover, they must be guided by a series of ethical values and principles. This involves, besides the rationalist analysis of alternatives, considering the ethical aspects of these alternatives. Aligning the interest of the healthcare unit with the patient’s interest often leads to ethical dilemmas in the managerial decision-making process, thus reflecting the sensitivity of the healthcare system [36].

### 4.4. Sources of Evidence in the Managerial Decision-Making Process Resulting from Research

The results of the content analysis of data collected through interviews are in line with the study by [37], which shows that there are six main sources of evidence used by hospital unit managers: (a) scientific evidence published in academic journals; (b) dashboards, the informational system as well as the internal evidence of the hospital unit; (c) socio-political development plans; (d) professional experience and judgment of hospital administrators; (e) ethics and religious beliefs; and (f) expectations of stakeholders.

Similarly, ref. [38] conducted a study in hospitals in the United States of America to examine whether healthcare unit managers use evidence-based management when faced with major decisions and what types of evidence they consult for decision making. The study results indicated that 90% of the participants responded that they had used evidence-based management for decision making in the last 6 months. Professional experiences (87%), organizational data (84%), stakeholder values (63%), and the opinions of colleagues/experts were the top four types of evidence consulted daily and weekly for decision making.

The use of evidence sources in decision making is supported through autoethnography in which the researcher, acting as a manager during the COVID-19 pandemic, made decisions regarding the procurement of drugs for treating COVID-19 patients using the SARS-CoV-2 infection treatment protocol issued by the Ministry of Health as the primary evidence source. When the unit he leads was designated a COVID-19 support unit, decisions were made regarding budget planning and resource allocation for purchasing equipment by firstly analyzing the specialized literature, European market catalogs, and international databases, and secondly the medical team members involved in treating COVID-19 patients. Considering that the unit he manages is a rehabilitation hospital, that specialized studies have demonstrated the presence of respiratory sequelae after infection with the SARS-CoV-2 virus, and that the number of patients presenting for post-COVID-19 rehabilitation was increasing, the researcher, in his capacity as a manager, decided to purchase special physiotherapy equipment for post-COVID-19 respiratory recovery. Furthermore, the researcher, in his role as a manager, decided to hire a psychologist to support both the COVID-19-infected patients and those presenting psychological sequelae post-COVID-19. In making this decision, the researcher relied on the specialized articles studied and the medical staff’s expertise. One of the major challenges faced by hospitals treating COVID-19 patients was the burnout syndrome among the medical staff, a phenomenon widely publicized by the press and observed in staff required to work for 8 h in protective, but not necessarily comfortable, equipment. In this context, the researcher, as a manager, decided to establish training and education centers within the unit to teach the medical staff how to best manage their working time in the COVID-19-contaminated red zone, to provide the best care to patients in that area, and at the same time to preserve their physical resources so necessary in this context.

Concluding the above, we can assert that the obtained data provided information to generate one of the research premises, namely that managers of hospital units in Romania make evidence-based decisions.

### 4.5. Managerial Decision-Making Models Resulting from Research

The results obtained from the analysis of the statements of the 10 interviewees regarding the use of managerial decision-making models are in accordance with the specialized literature. In this context, ref. [39] developed the administrative decision-making model to address the time constraints that exist in healthcare units. The model is largely based on a decision-making process, a hybrid of normative, descriptive, and naturalistic models that allows managers to make better decisions concerning clinical and administrative issues.

Ref. [40] defines the evidence-based model as decision making through the conscientious, explicit, and judicious use of four sources of information: the expertise and judgment of managers, evidence from the local context, critical evaluation of the best available research evidence, and the perspectives of the population. Furthermore, ref. [37] highlights the fact that the evidence-based model represents a guarantee of effective management.

Ref. [32] considers the adaptive decision-making model to be similar to what healthcare decision-makers actually do, more than evidence-based decision-making models, with the potential to emphasize flexibility and adaptability over rationality and reductionism.

Ref. [41] presents the DECIDE model as a decision-making model that describes a step-by-step decision-making process, created with the aim of assisting managers in hospital units.

Among the existing decision-making models, da Cunha Pacheco Junior and Gomes [42] consider that the incremental model is the best fit for the healthcare system. Throughout his activity as a manager, the researcher made numerous decisions using the evidence-based model, the DECIDE model, the administrative model, the adaptive model, as well as the incremental model.

When organizing the permanent supervision of the oxygen station, the decision was made using the administrative model. The researcher, acting as the manager of a hospital designated as a COVID-19 support facility, faced a situation where the continuous use of the oxygen station posed major risks to patient safety. In this context, he involved the stakeholders in solving the problem, taking their proposals into account to reach a consensus, which was subsequently communicated to the medical staff who had to observe the work environment, record this on the specific form, and immediately report any problems noticed, and to the technical staff who were organized in shifts so that they could constantly monitor the work station and take immediate action in the case of a malfunction.

When preparing the budget for medications, it was based on the evidence resulting from the analysis of consumption, as well as on the national catalog of prices for prescription human-use medicines. Additionally, a meeting of the steering committee and the drug committee was requested, during which the managerial board, together with the medical staff, analyzed the opinions and knowledge gained at conferences and congresses both in the country and abroad, the opportunity of acquiring new drugs, and the elimination or replacement of drugs that no longer comply with therapeutic protocols.

Moreover, when making changes to the organizational structure, this possibility was discussed in the steering committee and the Board of Directors, in order to obtain the approval of the local council. In this regard, the performance indicators from the unit’s dashboard, the evolution of migrant morbidity, as well as the Ministry of Health’s database were analyzed to see if there were specialized personnel in a certain field.

In the decision to reorganize activities following the handover of a building to the constructors, the researcher, acting as a manager, used the adaptive model. He faced incomplete information, time constraints, and resource limitations in which he had to weigh the costs and benefits of possible solutions from an institutional, ethical, economic, and clinical perspective and to establish priorities. After going through all the steps, the decision was made to keep all specialties, by reducing some activities or orienting services towards outpatient care and day hospitalization.

An example in which the researcher, acting as a manager, made decisions using the DECIDE model was during the pandemic when he faced the issue of staff redistribution. In this regard, he tried to maintain the quality of care for both COVID-19 and non-COVID-19 patients while simultaneously motivating the staff. In the first stage, he analyzed to see if he had employees with the skills for treating COVID-19 patients. Then, he reviewed the legislation to see if financial incentives were provided that he could communicate to the medical staff, later giving them the opportunity to volunteer. In this way, he managed to voluntarily redistribute skilled personnel to the COVID-19 red zone, who provided quality medical services, confirmed by the patients admitted and treated within the unit.

The incremental model was used when the hospital managed by the researcher was designated as a COVID-19 support facility by the Ministry of Health’s order. Essentially, this required organizing the unit to receive patients infected with the SARS-CoV-2 virus without having any other alternatives or certainties regarding the outcomes.

Political factors, such as adherence to Ministry of Health directives or alignment with local council approvals, often dictates operational boundaries, priorities, and resource allocations within hospitals. These factors can place pressure on managers to comply with top–down decisions while still ensuring ethical obligations towards patient safety, quality care, and staff welfare. Managers should use decision-making models like DECIDE or the adaptive model to balance political directives with ethical considerations, especially when addressing resource limitations or reallocating staff to new priorities. Hospital managers should foster transparent communications with all stakeholders to ensure that political constraints are addressed collaboratively and in line with ethical standards.

The conclusion emerging from the data obtained through interviews, specialized literature, and the events and experiences lived by the researcher as a manager is that the administrative model, the evidence-based model, the adaptive model, the DECIDE model, and the incremental model are all used in making various managerial decisions within public hospital units in Romania.

## 5. Conclusions

Through data triangulation using interview-based surveys and autoethnography, this study compared the researcher’s experience as a public hospital manager with data from interviews and the literature. This analysis identified key characteristics of the decision-making model in Romanian public hospitals, with managerial decisions often focusing on four areas: (a) budget and resource allocation, (b) recruitment and staff development, (c) technology acquisition, and (d) strategic planning.

Hospital decision making, more complex than in other organizations, is shaped by unique healthcare challenges, including personalized care, treatment complexities, system vulnerabilities, patient support needs, lack of clear hierarchy, and advanced technology [39]. These insights led to this study’s first premise: (P1) Unique aspects of Romanian hospitals influence managerial decisions.

This study also revealed that decisions are influenced by political, economic, moral, and ethical factors, leading to a second premise: (P2) Managerial decisions are shaped by external and ethical factors. Additionally, an evidence-based and consultative approach was identified, resulting in premises (P3) Managers make evidence-based decisions and (P4) Managers practice a consultative style.

Various decision-making models—including the administrative, evidence-based, adaptive, DECIDE, and incremental models—further delineate the specificities of decision making in Romanian public hospitals.

By outlining these characteristics and influencing factors, this study offers a framework not for prescriptive decision-making patterns but for supporting progress in hospital management to address increasingly complex challenges. This foundation can help guide improvements in managerial practices and performance within Romania’s healthcare system.

### 5.1. Research Limitations

The qualitative component of this study is constrained primarily by the potential influence of researcher biases on the analysis. This limitation arises from pre-existing assumptions and opinions, as well as the natural empathy that can develop between an interviewer and interviewee, which is a factor that may introduce risk in qualitative analysis [43]. Additionally, interviewee responses may be shaped by social desirability, leading them to answer questions in a favorable manner. To address this, the researcher adopted an open yet critical stance, engaging in active listening without providing feedback on the interviewees’ statements, thereby reducing the influence of such biases on data interpretation.

Another significant limitation pertains to the use of autoethnography as a complementary tool to reinforce interview findings. While autoethnography can deepen insights, it has been criticized in the literature for being “self-indulgent, narcissistic, introspective, and overly individualized” [11,44]. To enhance the reliability and validity of data collection, analyses, interpretations, and conclusions, data triangulation was employed, incorporating both analytical autoethnography and relevant scholarly literature. This approach aimed to mitigate limitations inherent to the qualitative methods and reinforce this study’s findings.

The sample of healthcare professionals interviewed is relatively small and geographically confined to the Northeastern region of Romania, which may limit the generalization of the findings.

### 5.2. Future Research Directions

This study offers relevance and utility to a broad spectrum of stakeholders, including researchers, academics, educators, doctoral candidates, hospital administrators, and health sector policymakers. Given its focus on the intricacies of managerial decision making in Romanian public hospitals, this work may serve as an important reference for public hospital management analysts, offering insights into the unique decision-making context within this sector in Romania.

An important avenue for future research involves using this study’s methodological framework to compare decision-making models in Romanian public hospitals with those implemented in countries recognized for exemplary hospital management practices. Such comparative analyses could reveal potential solutions to current national management challenges, highlight both convergences and divergences in decision-making approaches, and identify effective managerial decision-making patterns applicable across healthcare systems.

The strength of this study lies in its potential to generate fresh perspectives for future research by introducing new factors, exploring novel variables, or redefining existing ones within the managerial decision-making domain. This adaptability enables a comprehensive exploration of hospital management, unconfined to a single facet, thereby advancing a deeper and more nuanced understanding of effective managerial practices in healthcare.

## Data Availability

The data presented in this study are available on request from the corresponding author.

## Supplementary Materials

The following supporting information can be downloaded at https://www.mdpi.com/article/10.3390/healthcare12232395/s1. Supplementary Materials S1: Interview Guide. Supplementary Materials S2: Synthesis of Interviews. Supplementary Materials S3: Themes and subthemes of the “managerial decision-making” domain. Supplementary Materials S4: Themes and subthemes of the “elements of uniqueness of hospital units” domain. Supplementary Materials S5: Themes and subthemes of the “influencing factors of managerial decision” domain. Supplementary Materials S6: Themes and subthemes of the “sources of evidence in managerial decision-making” domain. Supplementary Materials S7: Themes and subthemes of the “managerial decision-making models” domain.
